# Exploring the Biological Value of Red Grape Skin: Its Incorporation and Impact on Yogurt Quality

**DOI:** 10.3390/foods13203254

**Published:** 2024-10-13

**Authors:** Eugenia Covaliov, Tatiana Capcanari, Vladislav Reșitca, Aurica Chirsanova, Alina Boiștean, Rodica Sturza, Antoanela Patras, Cristina Bianca Pocol, Olga Ruseva, Ana Chioru

**Affiliations:** 1 Department of Food and Nutrition, Oenology and Chemistry, Faculty of Food Technology, Technical University of Moldova, 9/9 Studentilor St., MD-2045 Chisinau, Moldova; tatiana.capcanari@toap.utm.md (T.C.); vladislav.resitca@adm.utm.md (V.R.); aurica.chirsanova@toap.utm.md (A.C.); alina.boistean@toap.utm.md (A.B.); rodica.sturza@chim.utm.md (R.S.); olga.ruseva@doctorat.utm.md (O.R.); ana.chioru@doctorat.utm.md (A.C.); 2 Department of Exact Sciences, Faculty of Horticulture, “Ion Ionescu de la Brad” Iasi University of Life Sciences, 3 Mihail Sadoveanu Alley, 700490 Iasi, Romania; antoanela.patras@iuls.ro; 3 Department of Animal Production and Food Safety, University of Agricultural Sciences and Veterinary Medicine of Cluj Napoca, 400372 Cluj Napoca, Romania; cristina.pocol@usamvcluj.ro

**Keywords:** grape skin, yogurt, phenols, antioxidant activity, syneresis, sensorial acceptance, CATA

## Abstract

The study was conducted to study the sustainability and enhanced nutrition gains obtained from incorporating grape skin powder (GSP) extracted from both Fetească Neagră and Rară Neagră grape varieties into yogurt. Grape skins are major leftovers from wineries, having high amounts of phenolic compounds and dietary fiber responsible for their ability to improve the characteristics of food. The research aimed to evaluate the effect of GSP addition at varying concentrations (0.5%, 1.0%, and 1.5%) on the yogurt’s physicochemical properties, antioxidant activity, color parameters, and sensory attributes. Analysis revealed that both Fetească Neagră and Rară Neagră GSP increased the total phenolic content and antioxidant activity; however, Fetească Neagră showed greater improvements, with TPC reaching 1.52 mg GAE/100 g and DPPH inhibition up to 26.63%. Although slightly lower, TPC rose to 1.43 mg GAE/100 g and DPPH inhibition increased to 18.93% with Rară Neagră enhancing these parameters conversely. Color changes were observed in fortified yogurts where lightness decreased (L*) and redness increased (a*) due to the pH-dependent anthocyanin stability. Syneresis, indicative of yogurt’s water-holding capacity, was reduced at higher concentrations of GSP from both varieties, suggesting improved textural integrity. Sensory evaluation indicated that consumers generally favored yogurts with lower concentrations of GSP. Yogurts fortified with Fetească Neagră GSP received higher overall preference, while those with Rară Neagră GSP were also well-received for their distinct flavor profiles when used at suitable levels. These results show that GSP from both types of grapes improves the nutritional value of yogurt and complies with the principles of sustainable food production through reutilizing agro-industrial waste.

## 1. Introduction

Viticulture and winemaking stand for an important branch that involves world economics not only because of its contribution to agricultural production but also due to cultural and economic impacts worldwide [1,2]. Around 7.3 m·ha of vineyard area produced 80.1 mt of fresh grapes in 2022, with an average yield of 11 t/ha. From this harvest, 50% (37.3 mt) was pressed for wine (34.1 mt) and musts or juices (3.2 mt), while 42% (31.5 mt) was used for table grapes and 8% (5.7 mt) for dried grapes [3,4]. The winemaking industry, closely tied to the agro-biotechnological cycle, generates significant waste—around 30% of the grape mass—comprising pomace, stalks, and wine lees, which can harm the environment if mismanaged [5]. On the other hand, the European Green Deal’s ‘Sustainable Products’ policy focuses on reducing material waste, highlighting a need for sustainable viticulture practices that are both ecologically and economically beneficial, addressing the entire value chain from production methods to waste management [6].

Increasingly, in the search for improved biological value of food products, one of the recent trends is to incorporate agro-food waste into food matrices [7]. This strategy not only seeks to eliminate surplus waste, but also sets out to deliver healthy food products. Various studies have been conducted using wastes from apple pomace [8,9], seed cakes from the oil industries [10,11,12], non-traditional plants [13,14], eggplant peel [15,16], potato peel [17], etc. Among these wastes, special interest has been given to grape pomace [18,19]. The potential of grape pomace lies not only in its functional properties but also in its rich composition of polyphenols, which can enhance the nutritional profiles of foods [20]. Research suggests that polyphenols can enhance the activity of beneficial gut bacteria, leading to improved gut microbiota balance [21]. Incorporating grape skin powder into food matrices increases the content of beneficial phenolic compounds such as quercetin, resveratrol, and rosmarinic acid. Quercetin, known for its anti-inflammatory and antioxidant properties, may enhance cardiovascular health, while resveratrol contributes to metabolic benefits and oxidative stress reduction [22,23]. Several works have demonstrated its positive action on the stability of food products [24,25,26] in addition to its protective action on human health [27,28]. This evidence underlines how grape pomace could turn into a valuable component in the formulation of functional foods that, in addition to waste reduction, will help improve health conditions.

The most commonly fortified food products with grape pomace include bakery items, pasta [29,30,31], sweets [32,33], dairy [34,35], and meat products [36]. Yogurt is a modern, popular, and nutritious dairy dessert, whose production has been increasing annually [37]. Yogurt is considered a healthy product largely due to its rich composition of nutrients and bioactive compounds. Moreover, yogurt contains live bacterial cultures, known as probiotics, which contribute to gut health by enhancing the gut microbiota, aiding digestion, and potentially bolstering the immune system [38]. In recent years, there has been a clear trend towards developing products that combine a dairy-protein base with various additives, including those of plant origin such as grains, vegetables, and fruits. This combination ensures a high level of nutritional balance, particularly in terms of vitamins and amino acid composition [39].

Considering the astringent issue within the global pollution caused by waste from industrial sector, several authors have studied the possibility of yogurt fortification with different extracts and compounds of food waste. Thus, carrot waste extracts [40], mango pulp fiber wastes [41], red cactus peel [42], compounds in red pepper waste [43], saffron floral waste [44], etc. have been mentioned to have a positive effect on the nutritional and technological properties of yogurt to fit within the circle of sustainability. This new approach might explore bioactive compounds and dietary fibers from food wastes to possibly provide enhanced antioxidant activities and higher dietary fiber levels in yogurt products [45].

Therefore, the aim of this research was to evaluate the biological value of red grape skin and to investigate the impact of its addition on yogurt, as part of a broader effort to enhance yogurt’s nutritional and technological profile while addressing sustainability goals through the utilization of grape pomace.

## 2. Materials and Methods

### 2.1. Materials

In yogurt formulations, pasteurized cow milk with 3.5% fat was used. In order to initiate the lactic fermentation, a lactic starter culture containing lactic acid bacteria with a concentration of ≥10^8^ CFU/g was used. This starter culture included *Streptococcus thermophilus*, *Lactobacillus delbrueckii* ssp. *bulgaricus*, *Lactobacillus acidophilus*, and *Bifidobacterium lactis*.

The grape pomace from the Fetească Neagră and Rară Neagră varieties, harvested in 2023 from the central region of the Republic of Moldova and used for yogurt fortification, was provided by the microvinification research center within the Technical University of Moldova, after grapes were crushed in order to obtain grape juice. The skin was manually separated from the stalks and seeds, then dried by convection at 50 °C with an air speed of 1 ms^−1^ until a moisture content of 7% was reached. The drying oven was connected to a computer equipped with software that registered the grape skin weight dynamics throughout the drying process. The targeted moisture level of 7% coincided with the point at which the weight of the grape skin powder stabilized, indicating that a constant mass had been achieved and ensuring the material’s suitability for subsequent analysis and fortification experiments. The dried grape skin was milled using a Kenwood cooking chef XL (Watford, UK) and sifted in order to obtain particles with 100 μm dimension. Afterwards, the grape skin powders were immediately vacuum sealed in plastic pouches to minimize exposure to oxygen and prevent degradation. These pouches were then stored at room temperature (21 ± 2 °C) in a dark environment to further protect the samples from light-induced degradation. This procedure was carefully adopted to maintain the integrity of polyphenolic and anthocyanin compounds until analysis. The timeframe from initial sample preparation to the completion of HPLC analysis did not exceed 90 days, ensuring that the possibility of compound deterioration was significantly reduced.

### 2.2. Methods

All the chemicals and reagents used in the research were analytical grade.

#### 2.2.1. Total Acidity and pH

Acidity of cow’s milk were determined as mentioned in standardized methods provided in international methods of AOAC method [46]. Total yogurt acidity was determined according to Ścibisz et al. [47] by potentiometric titration with NaOH 0.1 N to pH 8.2. The pH of yogurt was measured using a Benchtop pH Meter (inoLab^®^ pH 7110, Xylem Analytics, Weilheim, Germany). Obtained results were compared to values stipulated in national regulations [48].

#### 2.2.2. Proximate Composition

The moisture, protein, and glucide content of grape skin powders was assessed by according to AOAC Official Methods 925.10, AOAC Method 960.52, and AOAC SMPR^®^ 2018.001, respectively (Standard Method Performance Requirements (SMPRs^®^) for Sugars in Animal Feed, Pet Food, and Human Food) [49]. Total dietary fiber (TDF) content was determined based on AOAC Method 2017.16 [49] using a TDFC10 Dietary Fiber, Total, Assay Control Kit (Sigma-Aldrich Chemie GmbH, Steinheim, Germany).

#### 2.2.3. The Analysis of Polyphenols and Antioxidant Activity

The total polyphenol content was determined using Folin–Ciocalteu reagent, as described by Makkar [50] using a calibration curve with acid gallic, and expressed in mg Gallic Acid Equivalent (GAE) per 100 g product. This was performed after the hydroethanolic extracts (70% ethanol, 30 min, 38 ± 2 °C) were obtained from grape skin powders, by direct reading of the extract’s absorbance at 765 nm at a Shimadzu UV-1800 spectrophotometer (Shimadzu Inc., Kyoto, Japan). HPLC analysis was performed by a Waters 2695e Alliance HPLC system, coupled with a PDA Detector and the Empower^®^ 3 software (Waters, Milford, MA, USA). The column was a C-18 Waters XBridge (50 mm × 4.6 mm, 3.5 μm), with an injection volume of 20 μL and a flow rate of 0.7 mL/min. Mobile phase A: 0.1% trifluoroacetic acid (TFA) in water; mobile phase B: 0.1% TFA in acetonitrile. The chromatograms were monitored at 280 nm; compound identification was conducted using the retention time of standards and quantification using the peak area [51].

The free radical scavenging capacity of the grape skin powders in relation to the DPPH• free radical was assessed using a method adapted from Lin and Zhou [52]. The procedure involved reacting 0.1 mL aliquots of the analyzed samples with 3.9 mL of a 60 µM DPPH methanol solution, using methanol as the reference sample. To ensure accuracy, the samples were kept in the dark for 30 min, and absorbance readings were taken at one-minute intervals using a Shimadzu UV-1800 spectrophotometer (Shimadzu Inc., Kyoto, Japan) at a wavelength of 517 nm. These readings were used to construct kinetic curves detailing the interaction between the samples and the DPPH free radical solution. The results were reported as a percentage of DPPH inhibition.

#### 2.2.4. Anthocyanin Profile

The anthocyanins’ chromatographic profile was determined by HPLC analysis using the adapted OIV-MA-AS315-11 method [53], as explained by Taran et al. [54]. Instead of using formic acid and acetonitrile as the eluent, a mixture of orthophosphoric acid and acetone was employed, which allowed for better separation of anthocyanins across various groups. As a result, optimized conditions for separating anthocyanins were achieved using HPLC on an LC-20A Prominence chromatograph by Shimadzu, with a Hipersil ODS C18 column (5 µm, 4.6 mm × 150 mm) and an SPD-20AV UV/VIS detector (Shimadzu Inc., Kyoto, Japan). The quantification of individual monomeric anthocyanins was conducted using an internal standard method, with malvin chloride.

### 2.3. Yogurt-Making Procedure

The mass of yogurt was prepared using commercial pasteurized milk (3.5% fat) that was firstly mixed with grape skin powder of the two grape varieties in amounts of 0.5, 1.0 and 1.5% related to the total milk mass, then left for 2 h at room temperature (21 ±  2 °C) in order to facilitate the hydration process. The mixture was further inoculated with commercial starter culture (3%). Yogurt was fermented in a Euro Cuisine YM80 Electric Yogurt Maker (euro Cuisine, Bell Gardens, CA, USA) at 37 °C until the pH reached a value of 4.6 (approximately 6 h). Afterwards the samples were rapidly cooled to 14 °C and stored in the dark at 4  ±  2 °C.

### 2.4. Yogurt Proximate Composition

The yogurt proximate composition in terms of fat, protein, and dry matter content was according to Covaliov et al. [39].

### 2.5. Color Assessment

The impact of grape skin powder incorporation on yogurt color parameters was evaluated based on CieLab principles, described by Macdougall [55], using the Konica Minolta colorimeter CR-400 (Osaka, Japan). Chromatic components L* (Lightness), a* (redness) and b* (yellowness) were measured for each yogurt sample. Afterwards, the color difference ∆*E* was determined using Equation (1). Whiteness index was calculated according to Vásquez-Mazo et al. [56], using Equation (2).

(1) $$∆E=\sqrt{{\left(L_{sample}-L_{0}\right)}^{2}+{\left(a_{sample}-a_{0}\right)}^{2}+{\left(b_{sample}-b_{0}\right)}^{2}},$$

(2) $$WI=100-\sqrt{{\left(100-L\right)}^{2}+a^{2}+b^{2}}$$

### 2.6. Syneresis Index

Syneresis index was determined by the measurement of the quantity of the released serum (%) from yogurt matrix during storage. During the research time, samples at 1, 5, 10, 15, and 20 days of storage were taken in order to assess the syneresis.

### 2.7. Sensory Analysis

For the “Overall Liking of Yogurt” and “Consumer Test” analyses, the sensory evaluation was conducted in accordance with the international standard ISO 8586 [57]. An external recruitment process was used to assemble a panel of naive sensory assessors, without any particular criteria, to closely reflect the typical consumer demographic. The primary requirement for participation was the absence of lactose intolerance, ensuring all participants could fully engage in the evaluation. This approach was chosen to best determine consumer willingness to buy the product, aiming to capture authentic consumer preferences and reactions to the yogurt fortified with grape skin powder.

#### 2.7.1. Overall Liking of Yogurt

A consumer acceptance test was conducted with 113 participants (57.5% female, 42.5% male), aged between 18 and 52 years. Consumers were provided with a tasting sheet detailing the key quality parameters of the yogurt enriched with red grape skin. The evaluated attributes included appearance, color, texture, aroma, and taste, attribute mentioned in the National Regulations [48]. The yogurt was rated on a 9-point hedonic scale, ranging from 0 (“dislike extremely”) to 9 (“like extremely”).

#### 2.7.2. Consumer Test

The consumer acceptance was measured via panelists’ willingness to buy the new developed yogurt formulations as each panelist was asked to answer the question: Consider a scenario where you are at the grocery store and you notice a version of your regular yogurt made with grape skin addition available for purchase. Would you be inclined to choose it over the conventional yogurt you usually buy? Therefore, each consumer was asked to rank the yogurt samples, separately for each grape variety, from most to least liked, with the highest preference receiving a score of 1 and the lowest preference a score of 4.

#### 2.7.3. Check-All-That-Apply (CATA)

Evaluation of yogurt sensory parameters was carried out using the CATA questionnaire in line with methodology proposed by Biró et al. [58]. During the experiment, panelists were asked to check which sensory attributes were most suitable in each yogurt sample. Even though the sensory qualities of a product form the primary basis of its attributes, they can also include hedonic terms and emotional responses, as well as non-sensory properties, which is why terms like Surprised, Happy, Scarred, Acceptable, Indifferent, and Disgusted were introduced in the list. There was no restriction on the number of terms that assessors could choose. A panel of 15 trained assessors, reaching a consensus, developed a list of terms to describe the yogurt samples. These terms are detailed in Table 1.

### 2.8. Statistical Analysis

Statistical Analysis Differences between samples were assessed using analysis of variance (ANOVA) with a 5% significance level. CATA analysis was conducted with XLStat Software version 7.5.2 for Excel. All experiments were performed in triplicate, and the data were reported as mean ± standard deviation (SD).

## 3. Results and Discussions

### 3.1. Characteristics of Used Raw Materials

The quality of the used raw materials for yogurt preparation was assessed in terms of acidity for milk, whereas for the grape skin powder, its characteristics were evaluated in terms of protein, carbohydrates, total dietary fiber content, phenols, and anthocyanin profile (Table 2, Table 3, Table 4 and Table 5).

The acidity value for the pasteurized milk complies with national legislation, which mentions a value below 16 °T [48]. It is a well-documented fact that yogurt derives its value from the benefits lactic acid bacteria confer on the human digestive system [59,60]. On the other hand, plain yogurts that contain no additives do not have any biologically active compounds, fibers, or other enriching components. Due to this fact, many researchers have worked toward the nutritional and biological upgrading of yogurt [61,62].

Thus, by examining the parameter values presented in Table 2 for grape skin powder (GSP), it can be concluded that this by-product represents a valuable source of fiber and minerals, demonstrating significant potential for incorporation into food matrices. The protein content of GSP from both grape varieties ranges between 9.84% (Fetească Neagră) and 9.91% (Rară Neagră), which is notably higher than the 6.7% reported by Mendez et al. in their study on the potential of white grape skins [63].

Regarding fiber content, values ranging from 30.52% for Fetească Neagră to 31.21% for Rară Neagră were recorded. These findings contrast with those of Abdrabba and Hussein, who reported a protein content in red grape skin from Libya of 2.30%, which corresponds to 8.71% on a dry matter basis, while the total fiber content was 11.20%, equating to 42.42% dry matter [64].

### 3.2. Biological Value of Grape Skin Powder

The bioactive characteristics of GSP were evaluated by examining the polyphenol content, the profiles of anthocyanins and phenolic acids, and the ability to inhibit DPPH free radicals (Table 3).

The result showed that Fetească Neagră exhibited a TPC of 6.39 mg/g of powder, and Rară Neagră with 3.41 mg/g. Such a huge difference might be due to the fact that, in Fetească Neagră, phenolics occur in more complex forms or structures. Antioxidant activity, given as the percentage inhibition of the DPPH free radical, recorded higher values for Fetească Neagră with an inhibition percentage of 90.24%, than for Rară Neagră which expressed this inhibition as 69.01%. The obtained results confirm the observations of Lingua et al. [65] and Ky et al. [66], who stated that in many cases, a high content of phenolics goes hand in hand with an increased antioxidant activity.

Significant differences were highlighted in the phenolic profile of the grape skins from two red grape varieties (Table 4).

The analysis showed that Fetească Neagră has higher relative contents of most phenolic compounds compared to the varietal wine Rară Neagră. For example, a considerably high-level concentration of quercetin was noticed in Fetească Neagră (109.82 mg/100 g) in comparison to Rară Neagră (66.83 mg/100 g). Likewise, the quantity of rosmarinic acid was also much higher in Fetească Neagră (18.79 mg/100 g) than that of Rară Neagră (5.04 mg/100 g). Additionally, catechin and epicatechin, which are known for their strong antioxidant activity [67], were found in greater quantities in Fetească Neagră (4.75 mg/100 g and 1.04 mg/100 g, respectively) compared to Rară Neagră (0.86 mg/100 g and 0.50 mg/100 g, respectively). This observation supports the findings of Kyraleou et al. who indicated that these flavanols contribute significantly to the overall polyphenolic profile of grape skins [68]. Of particular note is that vanillin and syringic acid were also more concentrated in Fetească Neagră: 6.24 mg/100 g and 8.59 mg/100 g, respectively, compared to concentrations in Rară Neagră (4.43 mg/100 g and 5.56 mg/100 g). These phenolic acids are essential for their roles in the sensory attributes and stability of grape-derived products, as also discussed by several authors, who found that these compounds play significant roles in defining the flavor and aroma profiles of wines [60,69]. These findings are consistent with those reported by García-Beneytez et al. in their various studies, which concluded that grape varieties may differ significantly in their phenolic profile due to genetic and environmental factors [70,71]. On the other hand, Esparza et al. determined that climatic conditions during a given timeframe differently affected polyphenol synthesis in grapes stems, highlighting that factors beyond grape variety, such as environmental conditions, can significantly influence the phenolic composition of grape stem extracts, thus in grapes as well [72].

Anthocyanins have long been famed not only for their properties as pigments [73,74], but also for their potent antioxidant activities [75,76], which mitigate the effects of oxidative stress and reduce the risk of chronic diseases [77,78]. On the strength of this, interest in anthocyanins has been growing actively within the framework mechanisms of nutritional science and functional food [79,80]. The determination of the anthocyanin content of grape skins from Fetească Neagră and Rară Neagră varieties (Table 5) helps understand the application of these compounds in the enhancement of the food product’s nutritional profile.

Among the ten quantified anthocyanins, Malvidin-3-glucoside was predominant, constituting 63.84% of the total anthocyanin content in Rară Neagră grape skin powder (GSP) and 67.63% in Fetească Neagră. Significant differences were observed in the content of most anthocyanic compounds between the two grape varieties. For instance, petunidin-3-glucoside constituted 12.06% of the anthocyanins in Fetească Neagră GSP, whereas in Rară Neagră, it accounted for only 4.19% of the total anthocyanin content. Fetească Neagră also showed higher levels of delphinidin-3-glucoside, malvidol diglucoside, and peonidin-3-glucoside. Conversely, Rară Neagră GSP exhibited higher concentrations of peonidin-3-acetylglucoside, cyanidin-3-glucoside, malvidin-3-acetylglucoside, peonidin-3-coumarylglucoside, and malvidin-3-coumarylglucoside.

Similar findings, where malvidin-3-O-glucoside is identified as the predominant anthocyanin compound in red grape varieties, have been reported in the studies by García-Beneytez et al. [71] and Kyraleou et al. [68]. These studies corroborate the current data, highlighting malvidin-3-O-glucoside as a major contributor to the anthocyanin composition in red grapes, which underscores its significance in determining the pigmentation and potential health benefits of these grapes.

### 3.3. Impact of Grape Skin Powder on Yogurt Quality

#### 3.3.1. Yogurt Physicochemical Characterization

Compositional analyses in enhanced yogurt with grape skin powder (GSP) from Fetească Neagră and Rară Neagră (Table 6) showed several differences in the contents of moisture, proteins, fats, and acidity.

The incorporation of grape skin powder (GSP) into yogurt resulted in a slight decrease in moisture content, particularly in the FN1.5 and RN1.5 samples compared to the control. This decrease may be attributed to the interactions between GSP fibers and phenolic compounds, which possess water-binding properties that enhance the gel structure of the yogurt. While the additionally observed protein content showed a marginal increase with higher GSP concentrations, this enhancement in nutritional value was not significantly pronounced. Given that GSP is primarily composed of carbohydrates and fibers and is low in lipids, its impact on fat content remained minimal, with lipid composition staying relatively stable across all samples. Consequently, the addition of GSP, even at concentrations of 0.5%, 1.0%, and 1.5%, results in limited alterations to protein and fat levels, while potentially contributing to improved texture through its water-holding capacity.

These findings are aligned with previous research in the field. For example, Popescu et al. [81] fortified yogurt with apple pomace and reported similar impacts on compositional characteristics. Additionally, Varnaite et al. [82] and Stoica et al. [83] observed consistent results when cranberry or black carrot pomace powder was incorporated into yogurt, indicating modest changes in moisture, protein, and acidity levels. These parallel studies reinforce the notion that the addition of fruit and vegetable pomace can be integrated into yogurt to enhance its nutritional profile without significantly disrupting its fundamental composition.

#### 3.3.2. Total Phenols Content and Antioxidant Activity of Yogurt Fortified with GSP

**Figure 1:**
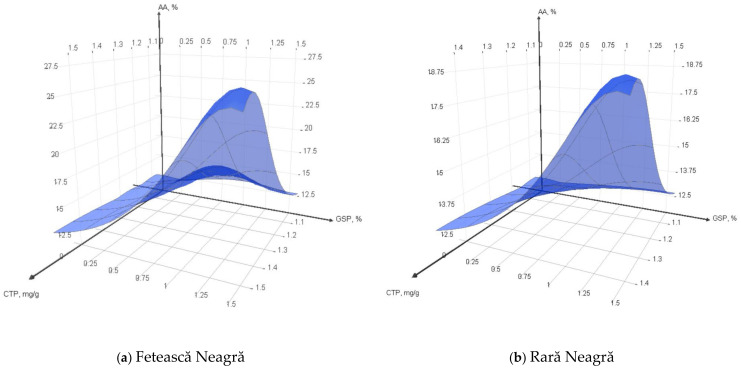
Impact of Fetească Neagră and Rară Neagră GSP concentration on total phenolic content and antioxidant activity of yogurt.

The addition of grape skin powder (GSP) from Fetească Neagră (FN) and Rară Neagră (RN) grape varieties to food products such as yogurt presents a promising approach to enhancing their bioactive properties. Figure 1a,b reveal the impact of varying concentrations of GSP on the total phenolic content (TPC) and antioxidant activity (AA), expressed as DPPH inhibition, to evaluate the potential nutritional benefits.

The obtained results proved that with increased concentrations of GSP, the TPC and AA in the samples increase. For the control sample values of 1.06 mg GAE/g and 12.4% were recorded for TPC and AA, respectively. When increasing the GSP to 1.5% concentration, FN showed a strong increase in TPC to 1.52 mg GAE/g, and a fairly strong increase in the AA to 26.63%. Meanwhile, RN showed a slightly lower increase in TPC to 1.43 mg GAE/g and a moderate rise in AA to 18.93%. This trend aligns with the results obtained by previous researchers, such as García-Lomillo and González-SanJosé who demonstrated a positive correlation between phenolic content enhancement and increased antioxidant capacity through the incorporation of grape pomace in food matrices [84]. Interestingly, beyond the quantitative enhancements of total phenolic content (TPC) and antioxidant activity (AA), recent studies have highlighted the superior bioavailability and bioactivity of polyphenols in fermented foods. According to Yang et al., the fermentation process enables the conversion of polyphenols into smaller, more absorbable phenolic compounds, such as quercetin and gallic acid. This suggests that the integration of grape skin powder into yogurt not only increases its phenolic content but also potentially enhances the effectiveness and health benefits of these compounds due to improved absorption and utilization in the body [85].

#### 3.3.3. Color Parameter Analysis

Color is one of the significant variables that consumers accept about yogurt. The control yogurt had an L* value of 81.25 and a whiteness index of 76.72, indicating normal unsupplemented appearance, while RN and FN GSP increased darkness in both yogurts, as can be seen by a significant reduction in L*. Among the samples, the RN1.5 sample had the lowest L* of 40.28 and *WI* of 39.80 and highest total color difference of Δ*E* of 44.96 (Table 7).

The chromatic components of the fortified yogurts also shifted, with a* values indicating an increase towards the red spectrum, notably in FN and RN samples. These changes could be attributed to the anthocyanins present in the grape skins, whose color expression is pH-dependent. According to the work of Arruda et al. [86], anthocyanins tend to become more red under acidic conditions, which is what the pH in yogurt was. The reduction in whiteness and the increase of Δ*E* of fortified samples highlight the intrinsic color contribution of GSP. Similar observations were made by Gomez Mattson et al. who evaluated the coloration changes in yogurt enriched with freeze-dried extracts from wild berries and noted significant darkening and hue shifts due to the interaction between polyphenolic molecules and the yogurt matrix [87].

#### 3.3.4. The Evolution of Yogurt Syneresis during Storage

Syneresis, the expulsion of whey from yogurt, is a critical quality attribute that reflects the stability of the protein matrix and overall textural integrity. The incorporation of grape skin powder (GSP) from Fetească Neagră (FN) and Rară Neagră (RN) was evaluated to assess its impact on yogurt syneresis over a storage period of 20 days, as indicated in Table 8.

The data reveal a complex interaction between GSP concentration and syneresis in yogurt. At a 0.5% concentration, GSP from both FN and RN appears to negatively impact the protein matrix, resulting in increased syneresis compared to the control. FN0.5 and RN0.5 yogurts exhibited slightly higher syneresis levels, with values reaching 29.34% and 28.92% respectively by the 20th day, compared to 28.06% for the control. This suggests that lower concentrations of GSP may interfere with the structural integrity of the yogurt matrix, potentially due to incomplete integration or uneven distribution within the protein network. In an antagonistic manner, samples fortified with 1.0% and 1.5% GSP demonstrated improved stability and reduced syneresis. The FN1.0 and RN1.0 samples showed a significant decrease in syneresis, with values of 26.04% and 26.22% respectively, indicating that higher concentrations may enhance the stabilization of the protein matrix. The FN1.5 sample, in particular, displayed the lowest syneresis at 24.48%, highlighting its potential as a stabilizing agent. García-Pérez et al. obtained similar results and stated that this fact is due to the high-water holding capacity of the fiber that absorbs the whey released by the gel structure [88]. Kumari et al., 2023 attributed this to the role of insoluble fiber fractions in trapping whey within the casein matrix, which helps to delay the occurrence of syneresis [89]. In the same vein, Mohamed Ahmed et al. attributes the reduction in syneresis in fortified yogurts to the interaction between polyphenols and milk proteins, which forms a firm gel network capable of retaining more water [90]. The behavior of the syneresis process in relation to GSP concentrations can be elucidated through the protein–polyphenol interaction model proposed by Siebert et al. According to this model, the binding sites of protein molecules for polyphenols are crucial for creating new networks that can limit whey separation from the gel. Specifically, our objective was to establish the balance between the number of available binding sites on the protein molecules and the concentration of polyphenols present in GSP. This interaction fosters the formation of stable structures that effectively retain moisture within the yogurt matrix, thereby reducing syneresis [91]. In the context of our findings, the 1.0% and 1.5% GSP concentrations enhance the protein–polyphenol interactions, resulting in the formation of tighter gel networks. This structural reinforcement is significant, as it minimizes the extent of syneresis and improves the overall texture of the yogurt. The interaction of polyphenols with proteins in yogurt fortified with GSP has been observed to sufficiently strengthen the gel structure, corroborating findings from Siebert et al.

Concerning pH values, after 20 days of storage, the final pH values ranged from 4.35 for the RN1.5 sample and 4.42 for FN1.5 to 4.50 for the control, indicating minimal fluctuation and suggesting a stable acid–base environment conducive to maintaining anthocyanin stability in the yogurt samples [92].

### 3.4. Consumer Test and Sensory Analysis

**Figure 2:**
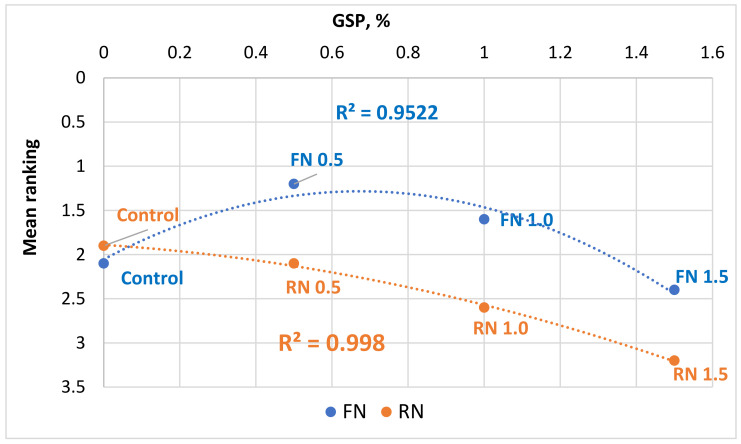
Mean ranking of yogurt enriched with different grape skin powder level. Control—control sample, FN0.5—yogurt with 0.5% addition of Fetească Neagră grape skin powder, FN1.0—yogurt with 1.0% addition of Fetească Neagră grape skin powder, FN1.5—yogurt with 1.5% addition of Fetească Neagră grape skin powder, RN0.5—yogurt with 0.5% addition of Rară Neagră grape skin powder, RN1.0—yogurt with 1.0% addition of Rară Neagră grape skin powder, RN1.5—yogurt with 1.5% addition of Rară Neagră grape skin powder.

The mean appreciation scores of yogurt samples at increasing GSP levels are shown in Figure 2. The panelists were asked to rank yogurt samples, separately for each GSP variety, according to their preference, from the most appreciated (score = 1) to the least appreciated (score = 4).

Figure 2 illustrates that the Fetească Neagră grape skin–fortified yogurts, were in general liked more than those fortified with the Rară Neagră GSP. In particular, the samples containing 0.5% and 1.0% Fetească Neagră GSP somehow exceeded even the control sample in preference, and were rated first and second, respectively, among yogurt samples with Fetească Neagră GSP.

Conversely, in the case of the Rară Neagră GSP enriched samples, a negative correlation emerged between the GSP level and the acceptability of the product; the lower the GSP content, the more appreciated yogurt was. It is clear that GSP, particularly from the Fetească Neagră variety, can be used in yogurt processing commercially; however, it is also apparent that GSP should be limited and not exceed 0.5% total without the change of such product acceptance by consumers. The preference for yogurts fortified with lower concentrations of grape skin powder (GSP) is likely influenced by several sensory attributes. At higher GSP concentrations, the fortified yogurts exhibited increased astringency and changes in texture, such as greater grittiness and a thicker mouthfeel, which was less appealing to consumers. Thus, the balance between enhancing nutritional value and maintaining desirable sensory qualities is crucial, and our results suggest that a lower GSP concentration, such as 0.5%, achieves this balance effectively. This highlights the potential for utilizing grape pomace as a functional ingredient in dairy products, as a novel functional ingredient focusing on additional product value without compromising on the requirements of the customers. The addition of GSP into yogurt improves the chemical and taste characteristics and helps achieve the goals of a circular economy and sustainability framework by cutting down on waste generated from farming activities and utilization of waste products in food processing industries.

**Figure 3:**
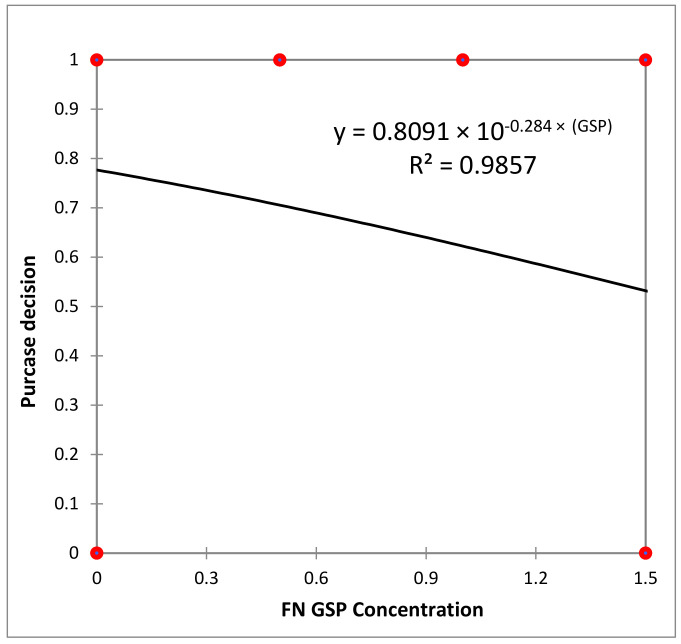
Logistic regression of yogurt purchase decision by Fetească Neagră GSP concentration.

**Figure 4:**
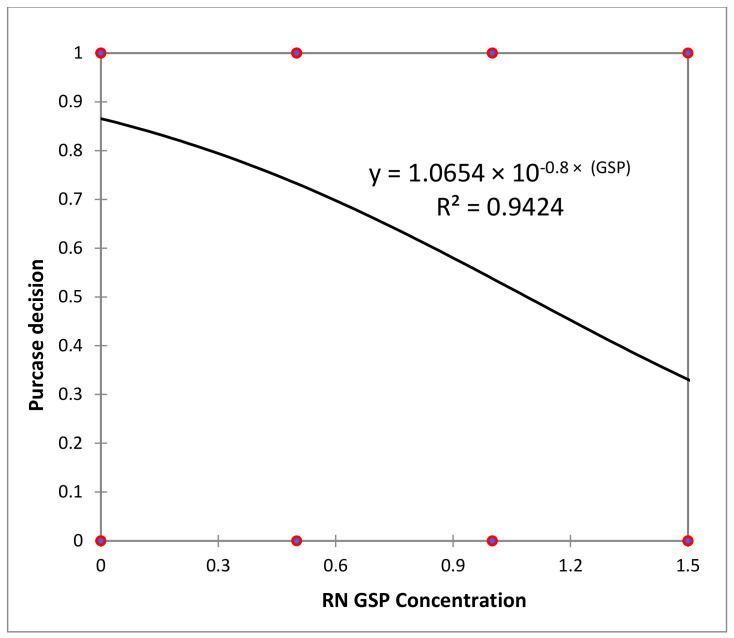
Logistic regression of yogurt purchase decision by Rară Neagră GSP concentration.

Concerning the consumers’ willingness to purchase (WP) each yogurt type, binary logistic regression was used to analyze the answers (Figure 3 and Figure 4).

The graphical representations of willingness to purchase (WP) as a function of grape skin powder (GSP) concentration (Figure 3 and Figure 4) indicates that over 75 and 63% of consumers would choose yogurt containing 0.5% and 1.0% Fetească Neagră GSP respectively. In contrast, for yogurt with Rară Neagră GSP, a similar trend as for the abovementioned ranking was observed, as these samples were less favored. Specifically, the proportion of consumers willing to purchase these products was 73%, 53%, and 34% for samples RN0.5, RN1.0, and RN1.5, respectively.

The check-all-that-apply (CATA) questionnaire originally included 36 properties terms (Table 1). After analysis of the consumers’ answering, it appeared that despite broad structure, several terms were selected infrequently, which meant that they were unnecessary for assessing the yogurt spiked with red grape skin. Among them, creamy white, earthy aroma, bitter taste, dry mouthfeel, gritty texture, scarred, and disgusted were the least selected choices.

**Figure 5:**
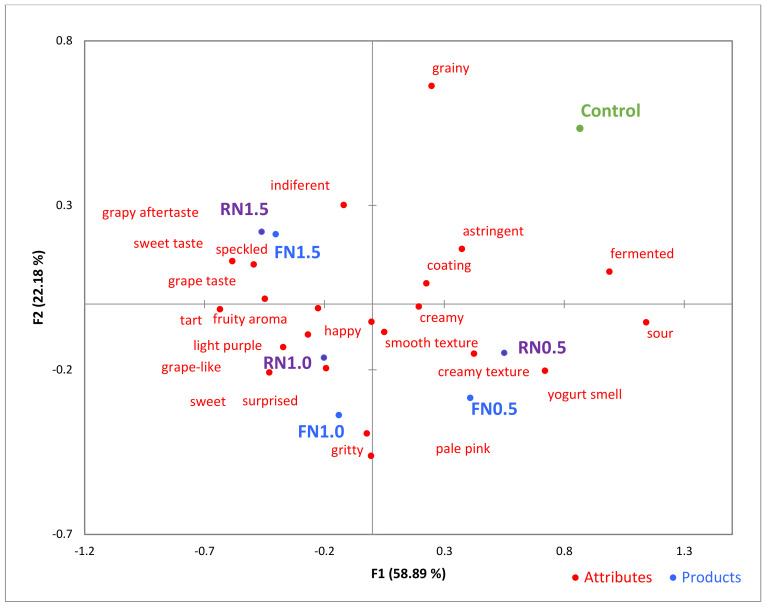
Visualized results of the check-all-that-apply (CATA) analysis of the seven yogurt samples. Control—control sample, FN0.5—yogurt with 0.5% addition of Fetească Neagră grape skin powder, FN1.0—yogurt with 1.0% addition of Fetească Neagră grape skin powder, FN1.5—yogurt with 1.5% addition of Fetească Neagră grape skin powder, RN0.5—yogurt with 0.5% addition of Rară Neagră grape skin powder, RN1.0—yogurt with 1.0% addition of Rară Neagră grape skin powder, RN1.5—yogurt with 1.5% addition of Rară Neagră grape skin powder.

Terms, including the above-mentioned ones, that did not follow with the objectives of the study such as included Cochran’s Q test as well in hugging the samples afforded by missing margins of the scores were removed so more accurate sensory analysis could still be attained. In doing so, we tried to take advantage of the term ‘25 CATA terms’ that were most useful, rather than looking into all the CATA terms that would be less preferred (Figure 5). This set of terms ensured that the sensory properties of our yogurt were properly and sensibly evaluated.

GSP addition from the FN and RN varieties had significant effects on the sensory properties of yogurt. When used in equal concentrations, such as 0.5% FN and 0.5% RN, the yogurt samples exhibited similar sensory attributes. This is evident from their proximity in the CATA plot (Figure 5), indicating that both grape skin powders impart comparable characteristics to the yogurt at this concentration level. The general features observed in the control samples, devoid of GSP, were creamy white color, smooth, mild odor of yogurt with light sweetness, creamy in mouthfeel, and neutral aftertaste.

After the addition of 0.5% GSP, the yogurt exhibited a pale pink color and developed a creamy texture with slight graining in yogurt samples. The yogurt had a fruity aroma with a hint of grape, a sweet-sour flavor profile, and provided a creamy mouthfeel with slight grittiness; it left a sweet aftertaste with a hint of sourness and elicited active and positive emotions. In contrast, yogurt samples containing 1.0% displayed a vibrant light purple hue and a thicker texture with more noticeable grittiness. These samples had a stronger grape aroma, a coconutty taste, a deeper grape flavor, a thicker mouthfeel, increased tartness, and a pronounced coconut aftertaste. The 1.5% samples received contradictory responses from consumers: some enjoyed the rich grape taste, while others disapproved of the excessive sourness.

## 4. Conclusions

The study emphasizes the significant potential of grape skin powder (GSP), derived from Moldovan Fetească Neagră and Rară Neagră grape varieties, as a valuable ingredient for enhancing the nutritional and technological attributes of yogurt. The results revealed that grape skin is rich in beneficial nutrients, including a protein content of 9.84% for Fetească Neagră and 9.91% for Rară Neagră, alongside impressive dietary fiber levels of 30.52% and 31.21%, respectively. Moreover, the total phenolic content of the grape skin powders was notably high, with Fetească Neagră exhibiting 6.39 mg GAE/g and Rară Neagră 3.41 mg GAE/g, both contributing to elevated antioxidant activity.

Among the various phenolic compounds identified in grape skin, Fetească Neagră had significantly higher relative levels of favorable compounds, including quercetin (109.82 mg/100 g) and resveratrol (4.37 mg/100 g), compared to Rară Neagră, which contained 66.83 mg/100 g of quercetin and 2.86 mg/100 g of resveratrol. The anthocyanin profile further illustrated the richness of these grape skins; for instance, Malvidin-3-glucoside, the predominant anthocyanin, made up 67.63% of the total anthocyanin content in Fetească Neagră, while it constituted 63.84% in Rară Neagră.

When incorporated into yogurt, grape skin substantially enhanced the total phenolic content, which increased from 1.06 mg GAE/100 g in the control sample to 1.52 mg GAE/100 g at a 1.5% concentration of Fetească Neagră, and 1.43 mg GAE/100 g for Rară Neagră yogurt sample with the same concentration. In terms of antioxidant activity, the levels were observed to rise from 12.4% in the control yogurt to 26.63% and 18.93% for yogurt with Fetească Neagră and Rară Neagră respectively at the highest incorporation levels. However, sensory evaluations indicated that while yogurts containing up to 1.0% GSP of Fetească Neagră and Rară Neagră were well-received, with over 75% of consumers expressing a willingness to purchase the 0.5% and 1.0% grape skin formulations, higher concentrations resulted in diminished overall acceptability.

The color and textural attributes of the fortified yogurts underwent notable modifications, particularly the reduction in lightness values and the accompanying visual perception of color, largely due to the anthocyanins in the grape skins. Additionally, at 0.5%, the addition of grape skin powder stimulates syneresis, increasing whey separation. However, at higher concentrations of 1.0% and 1.5%, it effectively inhibits syneresis, enhancing gel network stability and addressing storage challenges.

Our findings suggest that grape skin powder can be effectively utilized in yogurt formulations for both its health benefits and its potential to reduce waste in the food industry. Specifically, the optimal inclusion level appears to be 1.0% for Fetească Neagră grape skin, facilitating desirable sensory characteristics and consumer acceptability while enhancing the nutritional profile of yogurt. This integration of grape pomace aligns with sustainability goals and highlights opportunities for further research into enhancing functional food products using agro-food waste, thus promoting a more circular economy in the food sector.

## Figures and Tables

**Table 1:** CATA analysis terms.

Product Attribute	CATA Terms
Color	pale pink, light purple, creamy white, speckled
Texture	creamy texture, smooth texture, gritty, thick, grainy
Aroma	fruity aroma, grape-like, yogurt smell, fermented, sweet aroma, earthy
Taste	sweet taste, tart, sour, bitter, grape taste, astringent
Mouthfeel	creamy, gritty, smooth mouthfeel, dry, coating
Aftertaste	sweet, tart, bitter, grapy aftertaste
Emotions	surprised, happy, scarred, acceptable, indifferent, disgusted

**Table 2:** Physicochemical indicators of milk and grape skin powder.

Indicator	Milk (Commercial, Pasteurized)	Fetească Neagră Grape Skin Powder	Rară Neagră Grape Skin Powder
Acidity, °T	14.9 ± 0.63	-	-
Fat content, %	3.5 *		
Crude protein content, %	2.8 *	9.84 ± 0.32 ^a^	9.91 ± 0.27 ^b^
Glucides, %	4.7 *	12.77 ± 0.16 ^b^	12.43 ± 0.25 ^a^
Fiber content	-	30.52 ± 0.53 ^a^	31.21 ± 0.34 ^b^
Ash		15.84 ± 0.27 ^b^	15.23 ± 0.46 ^a^

Results indicate the mean value of three independent assays and are expressed as mean ± standard deviation (SD); different letters ^a,b^ mean significant differences (*p* ˂ 0.05); * the values (%) were taken from the product label.

**Table 3:** Total phenolic content and antioxidant activity of grape skins from Rară Neagră and Fetească Neagră varieties.

GSP Variety	TPC, mg/g	AA, %
Rară Neagră	3.41 ± 0.02 ^a^	69.01 ± 0,18 ^a^
Fetească Neagră	6.39 ± 0.6 ^b^	90.24 ± 0.21 ^b^

Results indicate the mean value of three independent assays and are expressed as mean ± standard deviation (SD); different letters ^a,b^ mean significant differences (*p* ˂ 0.05).

**Table 4:** Identification and quantification of phenolic compounds in the grape skin powder, expressed in mg/100 g product.

Nr.	Phenolic Compound	Relative Content, mg/100 g	
		Rară Neagră	Fetească Neagră
1	Galic acid	0.13 ± 0.01 ^a^	0.42 ± 0.01 ^b^
2	3,4-Dihydroxybenzoic acid	0.60 ± 0.02 ^a^	1.61 ± 0.08 ^b^
3	4-Hydroxybenzoic acid	0.49 ± 0.01 ^a^	1.05 ± 0.09 ^b^
4	Vanillic acid	1.22 ± 0.03 ^a^	1.60 ± 0.04 ^b^
5	Catechin	0.86 ± 0.02 ^a^	4.75 ± 0.11 ^b^
6	Chlorogenic acid	3.06 ± 0.09 ^a^	4.79 ± 0.17 ^b^
7	Vanillin	4.43 ± 0.14 ^a^	6.24 ± 0.21 ^b^
8	Syringic acid	5.56 ± 0.17 ^a^	8.59 ± 0.23 ^b^
9	Epicatechin	0.50 ± 0.01 ^a^	1.04 ± 0.02 ^b^
10	Ferulic acid	0.04 ± 0.01 ^a^	0.36 ± 0.01 ^b^
11	Sinapic acid	0.16 ± 0.01 ^a^	0.94 ± 0.02 ^b^
12	Resveratrol	2.86 ± 0.08 ^a^	4.37 ± 0.12 ^b^
13	Rosmarinic acid	5.04 ± 0.11 ^a^	18.79 ± 0.31 ^b^
14	Quercetin	66.83 ± 0.76 ^a^	109.82 ± 1.11 ^b^

Results indicate the mean value of three independent assays and are expressed as mean ± standard deviation (SD); different letters ^a,b^ mean significant differences (*p* ˂ 0.05).

**Table 5:** Identification and quantification of anthocyanin compounds in the grape skin powder, expressed in %.

Anthocyanin Compound	Relative Content, %	
	Fetească Neagră	Rară Neagră
Delphinidin-3-glucoside	5.96 ± 0.11 ^a^	2.09 ± 0.08 ^b^
Cyanidin-3-glucoside	0.54 ± 0.02 ^a^	0.89 ± 0.06 ^b^
Malvidol diglucoside	1.98 ± 0.14 ^a^	0.49 ± 0.05 ^b^
Petunidin-3-glucoside	12.06 ± 0.18 ^a^	4.19 ± 0.11 ^b^
Peonidin-3-glucoside	5.36 ± 0.09 ^a^	3.99 ± 0.14 ^b^
Malvidin-3-glucoside	67.63 ± 0.21 ^a^	63.84 ± 0.08 ^b^
Peonidin-3-acetylglucoside	0.54 ± 0.01 ^a^	0.71 ± 0.05 ^b^
Malvidin-3-acetylglucoside	2.34 ± 0.09 ^a^	6.07 ± 0.24 ^b^
Peonidin-3-coumaringlucoside	1.79 ± 0.02 ^a^	4.35 ± 0.09 ^b^
Malvidin-3-coumaringlucoside	3.75 ± 0.07 ^a^	13.35 ± 0.11 ^b^

Results indicate the mean value of three independent assays and are expressed as mean ± standard deviation (SD); different letters ^a,b^ mean significant differences (*p* ˂ 0.05).

**Table 6:** Quality parameters of yogurt samples.

Sample	Moisture Content, %	Protein Content, %	Fat Content, %	Acidity, % Lactic Acid	pH (after Fermentation)
Control	86.79 ± 1.12	3.21 ± 0.01	3.31 ± 0.12	0.46 ± 0.01	4.62 ± 0.03
FN0.5	86.75 ± 0.78	3.28 ± 0.02	3.28 ± 0.09	0.51 ± 0.01	4.53 ± 0.01
FN1.0	85.94 ± 1.05	3.33 ± 0.01	3.34 ± 0.13	0.49 ± 0.02	4.51 ± 0.02
FN1.5	85.37 ± 1.22	3.36 ± 0.03	3.30 ± 0.07	0.54 ± 0.01	4.50 ± 0.02
RN0.5	86.87 ± 0.83	3.27 ± 0.03	3.35 ± 0.11	0.48 ± 0.03	4.56 ± 0.01
RN1.0	86.05 ± 1.17	3.36 ± 0.01	3.33 ± 0.14	0.52 ± 0.01	4.48 ± 0.01
RN1.5	85.43 ± 1.24	3.36 ± 0.04	3.36 ± 0.09	0.54 ± 0.01	4.47 ± 0.03

Results indicate the mean value of three independent assays and are expressed as mean ± standard deviation (SD). Control—control sample, FN0.5—yogurt with 0.5% addition of Fetească Neagră grape skin powder, FN1.0—yogurt with 1.0% addition of Fetească Neagră grape skin powder, FN1.5—yogurt with 1.5% addition of Fetească Neagră grape skin powder, RN0.5—yogurt with 0.5% addition of Rară Neagră grape skin powder, RN1.0—yogurt with 1.0% addition of Rară Neagră grape skin powder, RN1.5—yogurt with 1.5% addition of Rară Neagră grape skin powder.

**Table 7:** Chromatic parameters of yogurt samples.

Sample	L*	a*	b*	Δ*E*	*WI*
Control	81.25 ± 1.14 ^e^	−4.23 ± 0.01 ^a^	13.14 ± 0.09 ^g^		76.72 ± 1.23 ^f^
RN0.5	56.38 ± 0.89 ^c^	5.65 ± 0.23 ^d^	2.45 ± 0.01 ^d^	28.82 ± 0.56 ^b^	55.95 ± 0.85 ^d^
RN1.0	51.44 ± 0.57 ^b^	6.42 ± 0.21 ^de^	0.35 ± 0.01 ^c^	34.14 ± 0.18 ^c^	51.02 ± 0.78 ^c^
RN1.5	40.28 ± 0.63 ^a^	7.47 ± 0.35 ^e^	−1.21 ± 0.02 ^b^	44.96 ± 0.28 ^e^	39.80 ± 0.52 ^a^
FN0.5	67.65 ± 1.05 ^d^	2.14 ± 0.02 ^b^	5.43 ± 0.11 ^f^	16.88 ± 0.15 ^a^	67.13 ± 1.05 ^e^
FN1.0	49.58 ± 0.65 ^b^	4.63 ± 0.04 ^c^	3.56 ± 0.08 ^e^	34.25 ± 0.42 ^c^	49.24 ± 0.79 ^c^
FN1.5	47.15 ± 0.85 ^b^	4.78 ± 0.03 ^c^	−2.37 ± 0.03 ^a^	38.53 ± 0.24 ^d^	46.88 ± 0.63 ^b^

Results indicate the mean value of three independent assays and are expressed as mean ± standard deviation (SD); different letters ^a–g^ mean significant differences (*p* ˂ 0.05). Control—control sample, FN0.5—yogurt with 0.5% addition of Fetească Neagră grape skin powder, FN1.0—yogurt with 1.0% addition of Fetească Neagră grape skin powder, FN1.5—yogurt with 1.5% addition of Fetească Neagră grape skin powder, RN0.5—yogurt with 0.5% addition of Rară Neagră grape skin powder, RN1.0—yogurt with 1.0% addition of Rară Neagră grape skin powder, RN1.5—yogurt with 1.5% addition of Rară Neagră grape skin powder.

**Table 8:** Evolution of yogurt syneresis during storage, %.

Sample	Storage Time, Days				
	1	5	10	15	20
Control	23.11 ± 0.14 ^b^	24.32 ± 0.10 ^bc^	25.34 ± 0.14 ^b^	26.41 ± 0.10 ^b^	28.06 ± 0.14 ^c^
FN0.5	24.15 ± 0.09 ^c^	26.52 ± 0.15 ^c^	26.95 ± 0.10 ^c^	27.24 ± 0.08 ^c^	29.34 ± 0.14 ^d^
FN1.0	22.25 ± 0.13 ^ab^	23.28 ± 0.08 ^b^	23.68 ± 0.08 ^a^	24.52 ± 0.08 ^ab^	26.04 ± 0.10 ^b^
FN1.5	22.03 ± 0.06 ^ab^	22.75 ± 0.12 ^a^	23.22 ± 0.12 ^a^	23.84 ± 0.10 ^a^	24.48 ± 0.09 ^a^
RN0.5	24.23 ± 0.12 ^c^	25.71 ± 0.08 ^c^	26.06 ± 0.10 ^b^	27.05 ± 0.11 ^c^	28.92 ± 0.12 ^c^
RN1.0	21.94 ± 0.07 ^a^	22.85 ± 0.12 ^a^	23.32 ± 0.16 ^a^	24.56 ± 0.14 ^ab^	26.22 ± 0.08 ^b^
RN1.5	21.48 ± 0.12 ^a^	22.07 ± 0.12 ^a^	22.78 ± 0.12 ^a^	24.24 ± 0.12 ^ab^	25.04 ± 0.16 ^a^

Results indicate the mean value of three independent assays and are expressed as mean ± standard deviation (SD); different letters ^a–d^ mean significant differences (*p* ˂ 0.05). Control—control sample, FN0.5—yogurt with 0.5% addition of Fetească Neagră grape skin powder, FN1.0—yogurt with 1.0% addition of Fetească Neagră grape skin powder, FN1.5—yogurt with 1.5% addition of Fetească Neagră grape skin powder, RN0.5—yogurt with 0.5% addition of Rară Neagră grape skin powder, RN1.0—yogurt with 1.0% addition of Rară Neagră grape skin powder, RN1.5—yogurt with 1.5% addition of Rară Neagră grape skin powder.

## Data Availability

The original contributions presented in the study are included in the article, further inquiries can be directed to the corresponding author.
